# Experience of the patient regarding their safety in the hospital environment

**DOI:** 10.1590/0034-7167-2022-0512

**Published:** 2023-10-09

**Authors:** Ana Cristina Pretto Báo, Cassiana Gil Prates, Marcelo Prado Amaral-Rosa, Diovane Ghignatti da Costa, João Lucas Campos de Oliveira, Simone Coelho Amestoy, Ana Maria Müller de Magalhães, Gisela Maria Schebella Souto de Moura

**Affiliations:** I Universidade Federal do Rio Grande do Sul. Porto Alegre, Rio Grande do Sul, Brazil; II Hospital Ernesto Dornelles. Porto Alegre, Rio Grande do Sul, Brazil; III Universidade Federal do Rio Grande do Norte. Natal, Rio Grande do Norte, Brazil; IV Universidade Federal de Santa Catarina. Florianópolis, Santa Catarina, Brazil; V Universidade Federal do Vale do São Francisco. Petrolina, Pernambuco, Brazil

**Keywords:** Patient Participation, Patient Safety, Patient-Centered Care, Quality of Health Care, Nursing, Participación del Paciente, Seguridad del Paciente, Atención Centrada en el Paciente, Calidad de la Atención de la Salud, Enfermería, Participação do Paciente, Segurança do Paciente, Assistência Centrada no Paciente, Qualidade da Assistência à Saúde, Enfermagem

## Abstract

**Objectives:**

to analyze the factors that can impact patients’ experience concerning safety-related measures in the hospital setting.

**Methods:**

this qualitative, descriptive, and exploratory study was conducted with patients and their family members at a hospital in southern Brazil. Semi-structured interviews were carried out using the Critical Incident Technique between January and February 2022. The collected data underwent content analysis with the assistance of IRaMuTeQ software.

**Results:**

five patients, four family members, and three patient-family units participated in the study. The following categories emerged: “Patientprofessional interaction as a component of safe care,” “Recognition of safety protocols in the patient’s experience,” and “Safe care and the challenges in hospital care.”

**Conclusions:**

patient-professional interaction, communication, awareness of safety protocols, and the availability of the nursing team are factors that influence patients’ experience regarding the safety of their care during hospitalization.

## RESUMO

**Objetivos:**

analisar os elementos que podem influenciar a experiência dos pacientes em relação às ações relacionadas à segurança do cuidado no ambiente hospitalar.

**Métodos:**

estudo qualitativo, descritivo e exploratório, realizado com pacientes e familiares em um hospital do sul do Brasil. Foram realizadas entrevistas semiestruturadas, com base na Técnica do Incidente Crítico, entre janeiro e fevereiro de 2022. Os dados foram submetidos à análise de conteúdo, com apoio do software IRaMuTeQ.

**Resultados:**

participaram cinco pacientes, quatro familiares e três pacientes-famílias. Surgiram as categorias “Interação paciente-profissional como elemento do cuidado seguro”, “Protocolos de segurança identificados na experiência do paciente” e “O cuidado seguro e seus desafios na assistência hospitalar”.

**Conclusões:**

a interação paciente-profissional, a comunicação, a identificação de protocolos de segurança e a disponibilidade da equipe de enfermagem são elementos que influenciam a experiência dos pacientes em relação à segurança do cuidado durante a internação hospitalar.

## RESUMEN

**Objetivos:**

analizar los factores que pueden afectar la experiencia de los pacientes en relación con las medidas de seguridad en el entorno hospitalario.

**Métodos:**

este estudio cualitativo, descriptivo y exploratorio se llevó a cabo con pacientes y sus familiares en un hospital del sur de Brasil. Se realizaron entrevistas semiestructuradas utilizando la Técnica del Incidente Crítico entre enero y febrero de 2022. Los datos recolectados se sometieron a un análisis de contenido con la ayuda del software IRaMuTeQ.

**Resultados:**

participaron cinco pacientes, cuatro familiares y tres unidades paciente-familia. Surgieron las siguientes categorías: “Interacción paciente-profesional como componente de atención segura”, “Reconocimiento de protocolos de seguridad en la experiencia del paciente” y “Atención segura y los desafíos en la atención hospitalaria”.

**Conclusiones:**

la interacción paciente-profesional, la comunicación, el conocimiento de los protocolos de seguridad y la disponibilidad del equipo de enfermería son factores que influyen en la experiencia de los pacientes en cuanto a la seguridad de su atención durante la hospitalización.

## INTRODUCTION

For over twenty years, patient safety has become a prominent issue worldwide and is defined as the reduction, to an acceptable minimum, of the risk of unnecessary harm associated with healthcare^(1)^. The pivotal moment in history was the publication of the report “To Err Is Human: Building a Safer Health System” by the Institute of Medicine in 1999, which prompted the need for various reforms in healthcare systems^(2)^.

In the subsequent decade, the 2008-2009 work plan of the World Alliance for Patient Safety, proposed by the World Health Organization (WHO), emphasizes the initiative called “Patients for Patient Safety,” with the objective of ensuring that the patient’s voice becomes the foundation of the movement for healthcare safety^(3)^. In Brazil, the National Patient Safety Program (PNSP), established by the Ministry of Health, also advocates for patient involvement in their own safety^(4)^.

Despite the established policy assumptions, it is recognized that patient engagement in their own care presents challenges, both due to the nature of healthcare delivery - which heavily relies on human factors - and the necessity to standardize methodologies and practices that can involve patients in this process, with the aim of achieving the safety goal, as corroborated by a systematic review^(5)^, in addition to enhancing the patient’s experience with healthcare delivery.

The investigation of patients’ experience in healthcare delivery is increasingly becoming a part of studies conducted from an organizational perspective to enhance quality^(6,7,8,9,10,11,12)^. The patient experience is defined as the culmination of all interactions that patients undergo in a healthcare institution, shaped by the organizational culture, which influences patients’ perception^(13)^.

Consequently, the involvement of patients as a component of care in healthcare systems^(14)^ has been progressively acknowledged. In the pursuit of promoting a safe environment, the inclusion of families and patients as stakeholders committed to care is one of the elements that should be incorporated in local patient safety plans across different healthcare institutions^(15)^. However, their active participation is still being consolidated, which poses challenges for professionals^(16)^, as well as for patients, families, and healthcare services.

A study conducted in Spain with patients receiving chronic care demonstrated that measuring the patient experience can facilitate the reorientation of healthcare systems towards integrated and patient-centered care^(17)^. Systematic reviews also suggest that patients can be a valuable source of information, capable of identifying, among other aspects, errors and factors that can jeopardize their own safety^(18,19,20)^.

It is evident that there is a gap in the literature regarding studies focused on the patient’s experience regarding their safety and the elements that constitute safe care in the hospital environment, from the patient’s perspective^(11,16,21)^. A review study, which aimed to identify incidents, adverse events, and contributing factors identified by patients, their families, and caregivers in the practice of hospital care, indicated that patients are capable of recognizing incidents and adverse events in care. Therefore, their participation and contribution to initiatives aimed at improving the quality and safety of care should be encouraged^(21)^.

Hence, this research aims to address the following question: How do hospitalized patients perceive the potentially influential elements of their experience regarding safety in care?

## OBJECTIVES

The objective of this study is to analyze the elements that can influence patients’ experience regarding actions related to safety in healthcare in the hospital environment.

## METHODS

### Ethical aspects

The research adhered to ethical and legal principles and received approval from the Research Ethics Committee of Hospital Ernesto Dornelles, with the attached opinion. The consent process included the use of institutional data document and informed consent forms for patients and their families. Authorization for the use of interviewees’ voices was included in the aforementioned forms.

### Study Type

This is a qualitative study of exploratory and descriptive nature. Semi-structured interviews were conducted to explore patients’ perceptions of actions for safe care in the hospital environment. The reporting of this study follows the guidelines of the Consolidated Criteria for Reporting Qualitative Research (COREQ).

### Study Setting

The research was conducted in a private general hospital located in the southern region of Brazil. The hospital holds the Accreditation certification from the Joint Commission International (JCI).

### Data Collection and Organization

The participants included five patients, four family members, and three patient-family units who were admitted to one of the six clinical and surgical units. Since the research involved the use of a validated questionnaire specific to the healthcare area under investigation^(22)^, it was decided to conduct the research within the inpatient units. The number of participants was intentionally determined, with the plan to interview two participants per unit. The semi-structured interviews could be conducted by the patient, their family members (in case the patient was unconscious or unable to respond), or by both the patient and a family member together, representing the patient-family unit. In this study, the interview statements were coded with the letter P for participant, followed by the chronological order of data collection for all interviews, regardless of whether the respondent was a patient, family member, or patient-family unit. Data saturation guided the decision-making process, and it was successfully achieved as indicated by the repetition of records.

Regarding the inclusion criteria for patients, it was decided to invite those who had been hospitalized for two or more days, as long as they showed interest in sharing their experience and had the clinical condition to move to a private room for the interview within the inpatient unit. For the patients’ families, the criterion used was their interest in reporting their perceptions and the fact that they had served as the primary family members during most of the hospitalization period. This criterion was established considering the perspective that the family member had participated in the patient’s experience regarding their care. To extend the invitation, the unit’s census list, containing the patients’ names with their respective admission dates, was used as a basis, selecting the first male patient and the first female patient in each unit. The exclusion criterion was to identify any communication impediment or condition that prevented the participant from describing their perceptions.

Data collection was carried out through interviews based on a semi-structured script that guided the investigation of patients’ experiences related to the study subject. The interviews took place in January and February 2022, conducted by the lead researcher, and followed the Critical Incident Technique (CIT)^(23)^, with a duration of 20 to 50 minutes. This technique allows for exploring and describing participants’ perspectives on significant situations they have experienced, providing an understanding of both positive and negative behaviors, situations, and consequences^(23)^.

The CIT suggests that memories be stimulated through an initial statement: “Think about your hospitalization regarding care related to your safety.” Then, sufficient time was given to remember a significant situation experienced or observed. The interview was then guided by a set of questions, including examples such as: “What situation do you remember?”; “What did you notice in the behaviors of the people involved?”; “Why did you select this event?”; “What could have been different?”.

The interviews were recorded on a mobile device and transcribed verbatim. The audio recordings will be stored for five years on a pen drive in the possession of the lead researcher and will be deleted after that period.

### Data Analysis

After the verbatim transcription of the interview data, thematic analysis was conducted jointly^(24)^, with the support of the IRaMuTeQ software (*Interface de R pour les Analyses Multidimensionnelles de Textes et de Questionnaires*) to organize the corpus for analysis. This involved identifying themes and grouping them to form categories, following the steps of pre-analysis, exploration of the material, treatment of the obtained results, and interpretation.

IRaMuTeQ is a free and open-source software developed under a license for textual analysis^(25)^. Based on the interpretations of content analysis^(24)^, supported by the IRaMuTeQ software^(25)^, three final categories were formed.

## RESULTS

A total of 12 interviews were conducted in the study, involving five patients, four family members, and three patient-family units. The analysis corpus used in the software consisted of the participants’ interviews (n=12), which comprised 13,201 words (an average of 1,100 words per participant). The data saturation criterion^(26,27)^ was achieved based on the Hapax coefficient (4.13%). A higher Hapax coefficient indicates greater uniqueness of statements, and thus, it is considered satisfactory^(28)^.

To obtain the results, Factorial Correspondence Analysis (FCA) and Hierarchical Descendant Classification (HDC)^(25,29)^ were performed. In FCA, it is possible to observe the associations of dependency and independence between each intermediate category. The Cartesian plane allows visualizing the level of dependency or independence through the distances or proximities between the variables^(29)^. Therefore, dependency associations occur in two situations: i) categories in the same quadrant, and ii) categories close to the lines/columns. On the other hand, independence correspondences occur when the categories are in different quadrants^(29)^.

**Figure 1:**
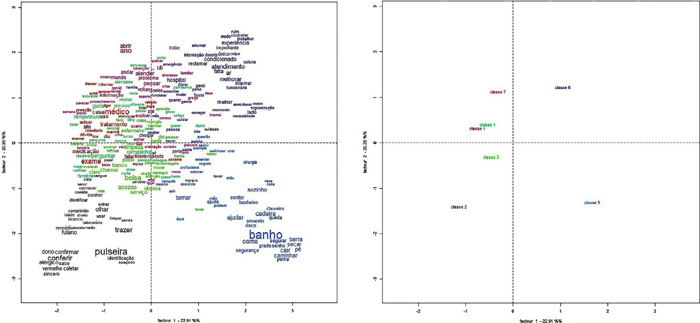
Factorial Correspondence Analysis of the words (in portuguese) in each intermediate category (left) and Factorial Correspondence Analysis of the intermediate categories (right)

Source: Data from IRaMuTeQ.

The Cartesian plane on the left (Figure 1) corresponds to the FCA of the words that compose each of the seven intermediate categories. It is possible to observe that the words vary in size according to their representativeness in each category. This graphical representation allows for the visualization of the interconnection and adherence of the terms ^(29)^, facilitating the interpretation of associations between the intermediate categories.

In the Cartesian plane on the right (Figure 1), we have the graphical representation of the FCA of the seven formed intermediate categories. Based on the spatial arrangement, there are seven types of associations between the intermediate categories, five of dependence and two of independence. Thus, it is possible to identify three levels of intensity for the associations: i) very low; ii) moderate; and iii) pronounced ^(30)^. When analyzing the associations between the intermediate categories (Figure 1), it is possible to observe the formation of seven sets.

**Figure 2:**
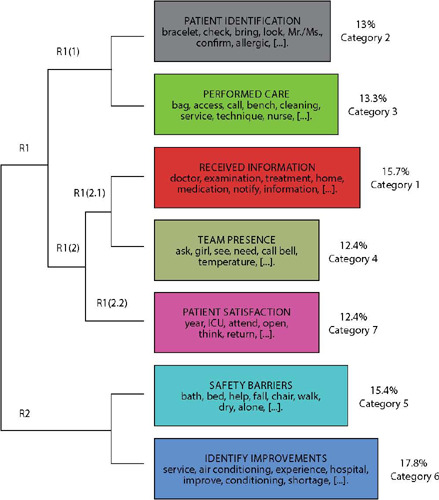
Hierarchical Descendant Classification dendrogram of the categories regarding the participants’ experiences

Source: Adapted data from IRaMuTeQ.

Another method used was the HDC analysis, which generated a dendrogram (Figure 2) with seven classes, following the standard nomenclature of IRaMuTeQ, which are considered intermediate categories in the Content Analysis method^(24)^. The intermediate categories are formed by approximations and distances of the text segments (TSs)^(26,28)^, according to the frequencies of the lemmatized occurrences of their vocabularies, until reaching the most stable configuration^(29,31,32)^. Each intermediate category in the dendrogram is composed of its main words. The classification of the TSs^(25)^ is based on the chi-square (χ2) statistical test^(29)^.

The titles of the seven intermediate categories were generated based on the interpretation of the meanings of the top 25 scoring TSs. Thus, the intermediate categories, their colors, and respective concentrations are: i) patient identification (gray - 13%); ii) performed care (green - 13.3%); iii) received information (red - 15.7%); iv) presence of the team (aquamarine - 12.4%); v) patient satisfaction (pink - 12.4%); vi) safety barriers (light blue - 15.4%); and vii) suggested improvements (blue - 17.8%). The higher the score value, the greater the density and importance of the TS within the intermediate categories^(26)^.

Based on the interpretations based on Content Analysis^(24)^, supported by the IRaMuTeQ software^(26)^, three final categories were formed.

### Patient-Professional Interaction as an Element of Safe Care

This category depicts the patient-professional interaction during hospitalization as an element of safe care, based on the shared experiences in the interviews. In the excerpts, the participants refer to the information received during their hospital stay. It is noted that information regarding treatment, medications, tests, and clinical results is provided by the medical professionals on a daily basis, as follows:

[...] *regarding the information for me and my family, the doctor would come here at 6 o’clock in the morning and tell me: we will have to stay a few more days, take medication, and we are doing tests.* (P4)

According to the participants, communication takes place during hospitalization and permeates various areas of the hospital, as evidenced in the following excerpt:

[...] *the doctors in the ICU also provided information, we would come to visit him, I would arrive at the ICU, and someone would come to accompany me to his bed, the nurses and doctors from the ICU would come to talk and explain everything to us.* (P1)

The healthcare team is also present in communicating with the patient at different times of the day. According to the accounts, nurses and nursing technicians are always available during different shifts, showing attentiveness and addressing the various needs of the patients, as stated in the excerpt:

[...] *the nursing technicians come to the room in all shifts, they ask how I am, if I’m in pain, if I need anything, and they say that if I need anything, I just have to call.* (P9)

On the other hand, despite receiving daily information, the following account reveals that there is no preparation for discharge regarding clinical treatment, medications, daily activities, and consequently, limited patient involvement in their own care, as stated below:

[...] *so tomorrow, when the doctor discharges me, he will provide information to me and my daughter, he will explain my treatment at home tomorrow upon discharge.* (P5)

### Safety protocols identified in the patient’s experience

In this category, we find the safety protocols perceived by the interviewees during the received care. It is noticeable that patients know the reason why they wear a white bracelet on their arm during hospitalization, as stated below:

[...] *I know that this bracelet is used to identify the patient* [...]. (P2)

Regarding the verification of the identification bracelet, an action that should be performed by professionals, the patient notices when it is carried out:

[...] *Every time they come here to administer medications; I notice that they* [nursing technicians] *look at the bracelet* [...]. (P1)

Another moment when the patient realizes that their name should be checked is during mealtimes. However, in the following statement, patients don’t perceive this verification:

[...] *those who deliver the food don’t check my name, they just drop off the tray and leave. I take care of what’s on the tray, my name with the printed paper, the type of diet. I always confirm it.* (P6)

Another safety protocol perceived by participants during hospitalization is the fall prevention protocol. Patients at high risk of falling receive a yellow bracelet. In their statements, although they don’t mention the presence of the signaling bracelet, they report safety measures adopted by the institution and care provided by healthcare professionals:

[...] *in the bathroom, there are handrails to hold onto, there’s a shower chair. I feel safe with their* [nursing technicians’] *help, I feel protected.* (P8)

In the following excerpt, the patient mentions that the instructions regarding the fall protocol during hospitalization came from the doctor, who even called the patient’s attention when they mentioned taking a shower alone:

[...] *don’t do that anymore because if you have a fall, your treatment can become complicated. So, there’s no reason for you to shower alone. The instructions about the risk of falling mainly came from the medical team.* (P4)

The interviewees also mention structural issues within the institution that can affect the fall prevention protocol. The lack of safety bars in some bathrooms and water leakage during showers are concerns perceived by the participants that generate some worry:

[...] *there are safety bars only on the toilet in that bathroom, so I prefer to shower while seated.* (P9)

[...] *the water from the shower doesn’t go into the drain, so when you take a shower, the water comes out near the room door, flooding the floor. I might slip and fall.* (P8)

### Safe care and its challenges in hospital care

Category 3 addresses the safe care provided by the healthcare team, as well as some challenges to be addressed in the institution.

The availability of the nursing team is evident in the participants’ statements. The nurses are always present in the rooms during different shifts. They make themselves available, clarify doubts, and ensure the review of the necessary care for good service. At the same time, the nursing technicians are involved in hygiene and comfort-related care, such as bathing, vital signs, and diaper changes, creating a sense of being well-assisted in the patient and their family:

[...] *the head nurses come here in the morning and at night, always. When they come, they say: if you need anything, just call me or ask for me, I’ll come. I have easy access to them.* (P11)

[...] *in the case of bathing, at first, I didn’t know how to handle it. The nursing technicians would come and give the bath, change the diaper.* (P1)

However, some challenges in providing care are perceived in the accounts. It is noticed that the patient and their family are still not sufficiently encouraged by the healthcare team to participate actively during hospitalization. The feeling of insecurity in the patient and their family leads them to be less involved in their care:

*The only thing I don’t do, which is the nursing technicians’ job, is emptying the bag, the colostomy bag, because I feel insecure. So, I ring the bell or ask when they come into the room.* (P1)

Another challenge is related to the structural aspects of the institution, with participants pointing out the lack of air conditioning in the hallways and a malfunctioning air conditioner in the room:

[...] *the healthcare professionals are attentive to all the care. The only thing I think could improve is the lack of air conditioning in the hallways, for the technicians and nurses to work better.* (P5)

[...] *but we stayed here for 40 days and changed beds, so it was terrible, terrible, terrible. They put us in a room where the air conditioning didn’t work, and the room was half the size of this one.* (P7)

The inadequate staffing levels in the nursing department were also noticed by the interviewees. While experiencing hospitalization, the patients and their families perceive the lack of staff and, at the same time, the efforts made by the team to provide good care:

*Since I arrived here, despite the pandemic and the lack of staff, the care is good. If I said it was bad, I’d be lying. It’s good, the nursing technicians make an effort.* (P7)

Despite pointing out some challenges and areas for improvement in the healthcare institution, the participants praise the care provided by the healthcare team. The care, dedication, attention, and affection shown by the healthcare team to the patient and their family during their unique experience instill confidence and credibility in the treatment received, as can be seen in the following excerpts:

*Well, what I remember from my father’s hospitalization, overall, it’s been very good. The people, the professionals, the doctors are very attentive.* (P2)

*Great hospital, wonderful professionals. I’m being well attended to. This experience I’m having is sensational.* (P5)

## DISCUSSION

The category Patient-professional interaction as an element of safe care proves to be an important element for patient safety, perceived in their experience. Communication, for the participants of the interviews, was present, a result that also appeared in a study^(33)^ carried out with patients and family members that identified attributes of satisfaction related to patient safety.

In addition, within this same category, the various moments in which communication takes place during hospitalization are described, according to the patient’s perception. Information received by the medical professional, the nursing team and the administrative staff, based on the presence of the team, and the interaction during the provision of care were listed as attributes of satisfaction.

It is noted, in the study, that patients emphasize daily information about their treatment, medications, examination and clinical results, received through the physician. Still, the appreciation of the medical professional is very present, but it is something that has been changing in hospitals, due to the implementation of a quality process, such as hospital accreditation^(34)^.

In the interviews, it was evidenced that, when needing to change sectors during hospitalization, the patient perceives the team’s concern in providing information, from the arrival at the hospital, in the emergency sector, to being transferred to an advanced care unit or even going to the ward.

Despite the existence of communication between patient and professional, it can be seen in the results that it is still necessary to improve the preparation of the patient for discharge, with regard to clinical treatment, the medications to be used and the activities allowed, in addition to the involvement of the patient in his care. Patients and family members feel prevented from getting involved in care processes or decision-making when they perceive that the team’s communication is disconnected and inadequate. However, when the family feels empowered to participate, the quality of patient care is enhanced^(35)^.

The involvement of the patient and his family, from arrival at the hospital to discharge, is an important option and can be thought of as a redesign of health care models, seeking to meet the needs of patients and improve health outcomes. For such a strategy, it is necessary, on the part of health professionals, to know about patient involvement in care safety. A study with health professionals reveals important aspects about the influence of patient involvement on care outcomes^(16)^.

The interaction between patient and nursing professional is perceived by the patient at different times and in different shifts of the day. Having their care demands met, when calling the nursing professional at the bell, such as checking vital signs, assessing pain and administering medication, makes the patient create a bond with the professional.

According to the present research, it is through the patienthealthcare professional interaction that patients identify satisfaction attributes regarding the care received. By listening to the patient, performing technical skills, treating them with care, providing support and attention, healthcare professionals can promote positive experiences and satisfaction for both the patient and their families, as well as contribute to patient loyalty to the healthcare institution.

A study^(36)^ found that patient satisfaction with the work of nurses in inpatient wards occurs through the use of technical skills, scientific knowledge, and individualization of nursing care. By viewing the patient as a unique and complex being, the professional enhances the planning and execution of care. Observing the individual needs of each patient and addressing them can substantially contribute to positive outcomes in terms of patient safety^(37)^ and can also make the hospital experience less traumatic and painful.

Our research also highlights, as an element of patient safety evaluation, the safety protocols perceived by patients and their families during the received care. In the second category, “Safety protocols identified in the patient’s experience,” the presence of the patient identification and fall prevention protocols was evident, although both are still in the process of consolidation in the institution. It is through the hospitalization experience that the patient and/or family identify elements that may be related to their safety. They feel secure when they perceive the presence of these safety measures, while also becoming concerned when they identify some weaknesses.

Patients acknowledge that they wear a bracelet for their identification and report moments when it is checked. The bracelet observed by the patient is white and contains identifiers such as the full name and date of birth, according to the Patient Safety Policy of the institution under study. Its use should occur to verify the patient’s identity before any procedure^(38)^.

The identification check is perceived by the patient at different moments of care. During medication administration, the patient notices that professionals check the identification bracelet to compare it with the medical prescription and also explain the purpose of a specific medication. Sometimes, the professional already knows the patient due to the length of hospitalization. Furthermore, the use of the bracelet seems to be a consolidated practice as it catches the patient’s attention when the device is not in good condition.

Another moment when the patient perceives the confirmation of their identification is during laboratory and imaging exams. When picking up the patient from their room, the professional verifies their data by calling them by name or looking at the identification bracelet. At the moment of receiving the diet, the patient does not perceive the verification of identification. The verification seems to be an action left to the responsibility of the patient themselves. In this situation, the patient identification verification protocol is still a challenge for the healthcare institution.

A study^(39)^ that reported the experience in the construction of a continuous quality improvement project for unequivocal patient identification also reinforced the idea of sensitizing the team. Unequivocal patient identification is essential to reduce incidents during care delivery. Therefore, strategies are necessary to raise awareness among professionals in implementing the patient identification procedure to ensure the quality and safety of care provided.

The patients also did not mention the yellow bracelet used by the institution for patients at risk of falls but pointed out measures adopted and perceived for fall prevention. However, they mentioned structural problems that can contribute to fall incidents.

In the interviews, the patients stated that they use wheelchairs or the available safety bars during bathing and adopt behaviors that make them feel safe and protected. At the same time, they reported receiving assistance from the nursing technicians for bathing. However, the instructions regarding the risk of falls were provided by the medical team.

It is known that safety protocols should be disseminated throughout the healthcare institution and practiced by all. A study conducted in a university hospital found the falls prevention protocol consolidated in the documents and in care practices. Fall prevention measures were present in the perceptions of patients and their families, as well as in the behaviors of professionals. Furthermore, it was noticed that professionals encourage the patient and the family to actively participate in care and preventive measures^(11)^.

The patients also mentioned problems related to the physical structure of the institution, such as the lack of safety bars and water leaks in the bathrooms. The perception of structural elements that can contribute to the patient’s fall demonstrates how attentive patients are and how their experiences can contribute to future improvements.

In the third category, “Safe care and its challenges in hospital care,” the effort of the care team to be present and attend to the patient is evident, but challenges to be addressed by the institution are also presented.

The availability of the nursing team in care related to bathing, comfort, diaper changing, vital signs monitoring, dressing changes, and concern for the patient’s well-being allows them to feel well-assisted. However, it is observed that the nursing team encourages the patient’s active participation in care very little, even though they have the potential for it.

Coproduction has been studied as a strategy in the pursuit of advances in patient safety^(11,40)^. In this sense, a study^(40)^ based on the patient’s experience presents the paths already taken and those to be taken in care coproduction. Coproduction was observed in some safety protocols, such as patient identification, fall prevention, and safe surgery. On the other hand, the lack of information hindered the follow-up of the treatment plan, resulting in cases of frustration for the patient. Based on their experience, the participants noticed the lack of air conditioning in the institution’s corridors or equipment failures in the rooms, as well as the inadequacy of the team’s staffing.

A study conducted in England highlights the importance of an adequate nursing team for patients. The results indicate that 57% of the patients perceive that there were not enough nurses to take care of them always or almost always, yet they rated the care as excellent. The patients’ perspective reinforces the importance of an adequate nursing team. It is also evident that the differences in this professional team in the evaluated hospitals are associated with neglected or forgotten nursing care, including post-discharge care management. It is suggested that appropriate bedside team staffing can be a promising strategy to increase patient satisfaction with care^(41)^.

Despite pointing out some challenges that can be addressed in the healthcare institution, the patients reported receiving care, attention, and affection from the care team, which aroused feelings of trust and credibility in them. A study^(40)^ shows that patient-centered care is perceived by patients and families, especially in moments of addressing health needs, such as bathing, medication administration, or diaper changing.

The study^(42)^ conducted with nursing managers showed the importance of managerial actions for the development of customer capital in organizations. For customer satisfaction evaluation, managers use their own assessment tools, active and systematic search, and feedback data. Customer satisfaction evaluation is not limited to the discharge moment or complaints from the feedback system. Different sources of information are used to capture and value the patient’s experience. Based on the customers’ desires, coordinated actions are involved to better serve and satisfy their needs.

It is known that in safe and trustworthy institutions, patients and their families are full members of the healthcare team as much as the professionals^(43)^. Therefore, their experience needs to be valued even more, causing true transformations in healthcare organizations. In this perspective, knowing the patient’s and their families’ views has been a priority, including to help build patient-centered care processes and improve the performance of clinical teams and organizations^(44)^.

Developing a study analyzing the patient’s experience regarding their safety allowed the elucidation of important results for the healthcare system. The patients have proven to be a source of information, capable of identifying elements that can compromise their own safety. In view of this, their protagonism, based on their involvement in care, can strengthen the safety culture of healthcare organizations.

### Study limitations

The study was conducted in only one hospital institution and took place during hospitalization, which may have contributed to the incomplete registration of the hospitalization experience. However, the Critical Incident Technique used to conduct the interviews provided memorable events in the hospitalization experience.

### Contributions to Nursing, Health, or Public Policy

This research contributes to expanding nursing knowledge about the patient’s experience and safety in the hospital environment, as well as highlighting the involvement of the patient and their family in their care. Furthermore, it is hoped that the results presented will support new scientific productions regarding the patient’s experience, their involvement in care, and proposals for care involvement models.

## CONCLUSIONS

Our study allows concluding that patients and their families identify elements related to care safety based on their experience, such as patient-professional interaction, communication, identification of safety protocols, and availability of the nursing team.

Additionally, some safety protocols were not perceived by the participants during their experience. Therefore, it is suggested that the healthcare institution, even working with hospital accreditation, can strengthen its safety culture by involving the patient and their families in planning improvements regarding patient safety.

Finally, despite patients and families presenting elements that can be considered as challenges by the institution, they express satisfaction with the care, leaving the institution with a positive perception of the experience.
